# SCORE2‐Older Persons (SCORE2‐OP): Validation and Added Value of Excessive Daytime Sleepiness in a French Cohort

**DOI:** 10.1111/jgs.70047

**Published:** 2025-08-28

**Authors:** Tahiana Andriambelosoa, Clémence Cavaillès, Jean‐Philippe Empana, Thibault Mura, Catherine Helmer, Yves Dauvilliers, Isabelle Jaussent

**Affiliations:** ^1^ Institute for Neurosciences of Montpellier (INM), Inserm University of Montpellier Montpellier France; ^2^ Department of Psychiatry and Behavioral Sciences University of California San Francisco San Francisco California USA; ^3^ Inserm, PARCC, F‐75015, Team Integrative Epidemiology of Cardiovascular Diseases Université Paris Cité Paris France; ^4^ Department of Biostatistics, Epidemiology & Public Health, CHRU Nîmes University of Montpellier Nîmes France; ^5^ Bordeaux Population Health Center, UMR U1219, Inserm University of Bordeaux Bordeaux France; ^6^ Sleep‐Wake Disorders Unit, Department of Neurology Gui‐de‐Chauliac Hospital, CHU Montpellier Montpellier France

**Keywords:** aging, cardiovascular diseases, primary prevention, risk assessment, sleep

## Abstract

**Background:**

Cardiovascular diseases are the leading cause of death worldwide, particularly in older adults. While the Systematic Coronary Risk Evaluation 2‐Older Persons (SCORE2‐OP) model estimates 10‐year cardiovascular risk in this population, its validation in general European cohorts remains limited. Given growing relationships between sleep disturbances and cardiovascular risk, we aimed to validate SCORE2‐OP in a French cohort and assess the incremental value of excessive daytime sleepiness (EDS).

**Methods:**

We included 4626 participants aged 70+ from the Three‐City cohort, without cardiovascular disease and dementia at baseline. The SCORE2‐OP model calibrated for low‐cardiovascular risk regions was used to estimate 10‐year cardiovascular risk. Calibration was assessed using observed‐to‐expected cumulative incidence ratios and calibration curves; discrimination using the area under the curve (AUC). Cox proportional hazards models examined associations between sleep symptoms (poor sleep quality, EDS, insomnia symptoms, sleep apnea [proxy]) and cardiovascular events. Incremental predictive value of significant symptoms was quantified by ΔAUC and net reclassification improvement (NRI).

**Results:**

Over 10 years, the observed cumulative incidence of fatal and non‐fatal coronary heart disease or stroke was 10.55%; 95% confidence intervals (CI) = (9.62; 11.48), while the SCORE2‐OP predicted risk was 14.04%; 95% CI = (13.85; 14.24), yielding an observed‐to‐expected ratio of 0.75; 95% CI = (0.69; 0.80). Discrimination was moderate (AUC = 61.71%, 95% CI = [58.64; 64.78]). EDS was the only sleep symptom independently associated with cardiovascular events (adjusted hazard‐ratio = 1.32, 95% CI = [1.05; 1.65]). Adding EDS to SCORE2‐OP did not improve overall discrimination (ΔAUC = +0.72%, 95% CI = [−0.05; 1.50]) or reclassification (NRI = +1.33%, 95% CI = [−3.27; 5.93]). Sex‐stratified analyses showed significant improvement in discrimination in men (ΔAUC = +2.27%, 95% CI = [0.54; 4.00]), but not in women. Moreover, EDS improved reclassification in low (< 7.5%) and intermediate (7.5%–15%) cardiovascular risk groups (NRI = +12%, 95% CI = [3.56; 20.43] and NRI = +12.44%, 95% CI = [7.23; 17.65], respectively).

**Conclusions:**

In this French cohort, SCORE2‐OP overestimated cardiovascular risk and showed moderate discrimination. EDS improved SCORE2‐OP performance in intermediate cardiovascular risk groups where treatments are uncertain, highlighting its clinical relevance; although implications for prevention strategies require further study.

## Graphical abstract

External validation of SCORE2‐OP in older French adults and incremental value of excessive daytime sleepiness for cardiovascular risk prediction: SCORE2‐OP showed moderate discrimination and overestimated risk by 25%. Adding EDS did not improve overall model performance but enhanced classification in low‐ and intermediate‐risk groups.

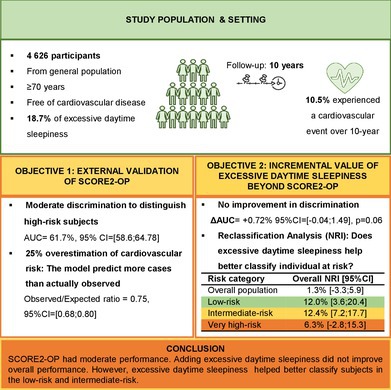

**Summary**

- Key points
  ○ Systematic Coronary Risk Evaluation 2‐Older Persons (SCORE2‐OP) overestimated the 10‐year risk of cardiovascular events and showed moderate discrimination between low‐ and high‐risk subjects.
  ○ Excessive daytime sleepiness was the only sleep symptom independently associated with cardiovascular events.
  ○ Although adding excessive daytime sleepiness to SCORE2‐OP did not improve overall model accuracy, it improved the reclassification of subjects at intermediate cardiovascular risk (i.e., with a 10‐year cardiovascular risk between 7.5% and 15%).
- Why does this paper matter?
  ○ This study validated the SCORE2‐OP in a French cohort of older adults and assessed the added value of sleep symptoms, specifically excessive daytime sleepiness, for cardiovascular risk prediction. These findings highlight the clinical relevance of excessive daytime sleepiness in improving cardiovascular risk stratification, especially in subjects at intermediate risk, for whom treatment decisions are often the most uncertain.

## 1 Introduction

Cardiovascular diseases (CVD) are the leading cause of death globally, accounting for approximately one‐third of all deaths, with 20.5 million in 2021 [1]. Beyond mortality, CVD contributes to significant loss of quality of life and excess costs to healthcare systems. Early identification of individuals at high cardiovascular risk is essential, especially in older adults [2]. Accurate cardiovascular risk prediction is important, especially in this population where comorbidities are frequent, requiring careful consideration of every therapeutic decision to limit the risk of overtreatment or undertreatment [3]. To address this need, cardiovascular risk prediction models have been developed to estimate individual risk of cardiovascular events. Among them, the Systematic Coronary Risk Evaluation 2‐Older Person (SCORE2‐OP), introduced in 2021, is specifically designed for adults aged 70+, predicting 10‐year fatal and non‐fatal cardiovascular risk using traditional risk factors (age, sex, cholesterol, smoking status, diabetes, and blood pressure) and accounting for competing non‐cardiovascular deaths [4].

Despite its potential, the external validity of the SCORE2‐OP remains limited. Most validations have been conducted outside Europe (e.g., ARIC, MESA) [5] or in specific clinical trial populations (e.g., HYVET, PROSPER, SPRINT) [5], and few have been done in general healthcare settings (e.g., CPRD [5], EMPRS [6], NHIP [7]). Only two external validation studies have been performed in general European populations at low cardiovascular risk (EPIC‐Norfolk [8] and Barcelona [9]), highlighting the need for further validation in similar settings.

In parallel, there is growing interest in improving cardiovascular risk prediction by integrating non‐traditional risk factors that are easily evaluated, inexpensive, non‐invasive, and clinically meaningful. Unhealthy sleep behaviors are relevant to investigate as they are recognized contributors to CVD [2, 10]. Epidemiological studies have shown that sleep disturbances, including excessive daytime sleepiness (EDS) [11, 12], insomnia [13, 14, 15], obstructive sleep apnea syndrome (OSAS) [16] and sleep duration (short and long sleep) [17] are associated with an increased risk of both fatal and non‐fatal cardiovascular events. The importance of sleep health in cardiovascular prevention has been further supported by the recent addition of sleep duration to the American Heart Association's Life's Essential 8, as well as the recognition of other key sleep dimensions such as its regularity, timing, and chronotype [18, 19]. Despite this evidence, no study has yet evaluated the added value of sleep symptoms in cardiovascular risk prediction models, including SCORE2‐OP.

This study aims to (1) externally validate the SCORE2‐OP model in a French cohort of older adults; and (2) assess the incremental value of sleep symptoms.

## 2 Methods

### 2.1 Study Population

We used data from the French population‐based Three‐City (3C) Study, which enrolled 9294 non‐institutionalized adults aged 65+ between 1999 and 2001 in Bordeaux, Dijon, and Montpellier [20], with follow‐up every 2–3 years for up to 14 years. For this analysis, we included 4626 participants aged 70+, free of baseline CVD and dementia, with complete data on SCORE2‐OP predictors, EDS, and at least one follow‐up for cardiovascular events (Supplement 1 in Supporting Information, Figure S1, Table S1).

All participants gave written informed consent. This study was approved by the KremlinBicêtre and Sud‐Méditerranée III ethics committees.

### 2.2 Cardiovascular Data Collection, Outcome Assessment, and Competing Events

At baseline, participants reported information on their history of coronary heart disease (CHD) and stroke. The primary outcome was the first non‐fatal cardiovascular event (CHD or stroke) or cardiovascular death over 10 years, consistent with the SCORE2‐OP development study [5]. Incident cardiovascular events were identified through follow‐up interviews, validated using medical records, and adjudicated by expert committees [21, 22]. Competing events were defined as non‐cardiovascular deaths (see Supplement 2 in Supporting Information).

### 2.3 Predictors of Cardiovascular Events

Traditional cardiovascular risk factors, including demographic characteristics, smoking status, diabetes, blood pressure, lipid profile, body mass index, and use of antihypertensive and lipid‐lowering treatments, were assessed at baseline using standardized protocols (see Supplement 3 in Supporting Information).

### 2.4 Sleep Symptoms

Baseline sleep symptoms were self‐reported, including EDS, insomnia symptoms (e.g., difficulty in initiating sleep [DIS], difficulty in maintaining sleep [DMS], early morning awakening [EMA]), loud snoring, and sleep quality. A composite Clinical Sleep Severity (CSS) score incorporated sleep quality, EDS, and insomnia symptoms [20]. The likelihood of sleep apnea was defined by frequent or often loud snoring combined with either EDS or poor sleep quality (definitions in Supplement 4 in Supporting Information).

### 2.5 Statistical Analysis

#### 2.5.1 External Validation of SCORE2‐OP

We externally validated the SCORE2‐OP using published sex‐specific equations calibrated for low‐risk European regions [4]. Calibration and discrimination evaluations accounted for competing risks (i.e., non‐cardiovascular deaths). Calibration was evaluated using observed‐to‐expected (*O*/*E*) ratios and calibration curves based on deciles of predicted risk. Discrimination was assessed by time‐dependent AUCs at 2, 4, 6, and 10 years. Sex differences analyses were performed using bootstrap procedures [21].

#### 2.5.2 Added Value of Sleep Symptoms to SCORE2‐OP

We assessed associations between sleep symptoms and incident cardiovascular events using multivariable Cox models. The proportional hazards assumption was tested using Schoenfeld residuals. Sleep symptoms significantly associated with incident cardiovascular events were further tested for their added predictive value. Only EDS showed a significant association and was added to SCORE2‐OP in a Fine and Gray model (SCORE2‐OP‐EDS). Its incremental value was evaluated by changes in discrimination (ΔAUC) and risk reclassification (NRI) (see Supplement 5 in Supporting Information). Analyses were performed using R 4.4.1 and SAS 9.4.

## 3 Results

### 3.1 Study Sample

At baseline, participants had a mean age of 75.9 (SD = 4.4) and 63.0% were females. Over 10 years, 392 (8.5%) experienced a non‐fatal cardiovascular event (11.3% in males, 6.8% in females). Additionally, 74 (1.6%) died from cardiovascular causes (2.5% in males, 1.1% in females) and 766 (16.6%) died from non‐cardiovascular causes (23.3% in males, 12.6% in females).

Compared to the subjects in the SCORE2‐OP development study (CONOR, a Norwegian cohort used to develop the SCORE2‐OP [5]), 3C participants were older, more often female, non‐smokers, diabetic, and took more frequently lipid‐lowering drugs. Furthermore, they had lower SBP and total cholesterol but higher HDL‐c levels (Table S2).

### 3.2 External Validation of the SCORE2‐OP

**FIGURE 1:**
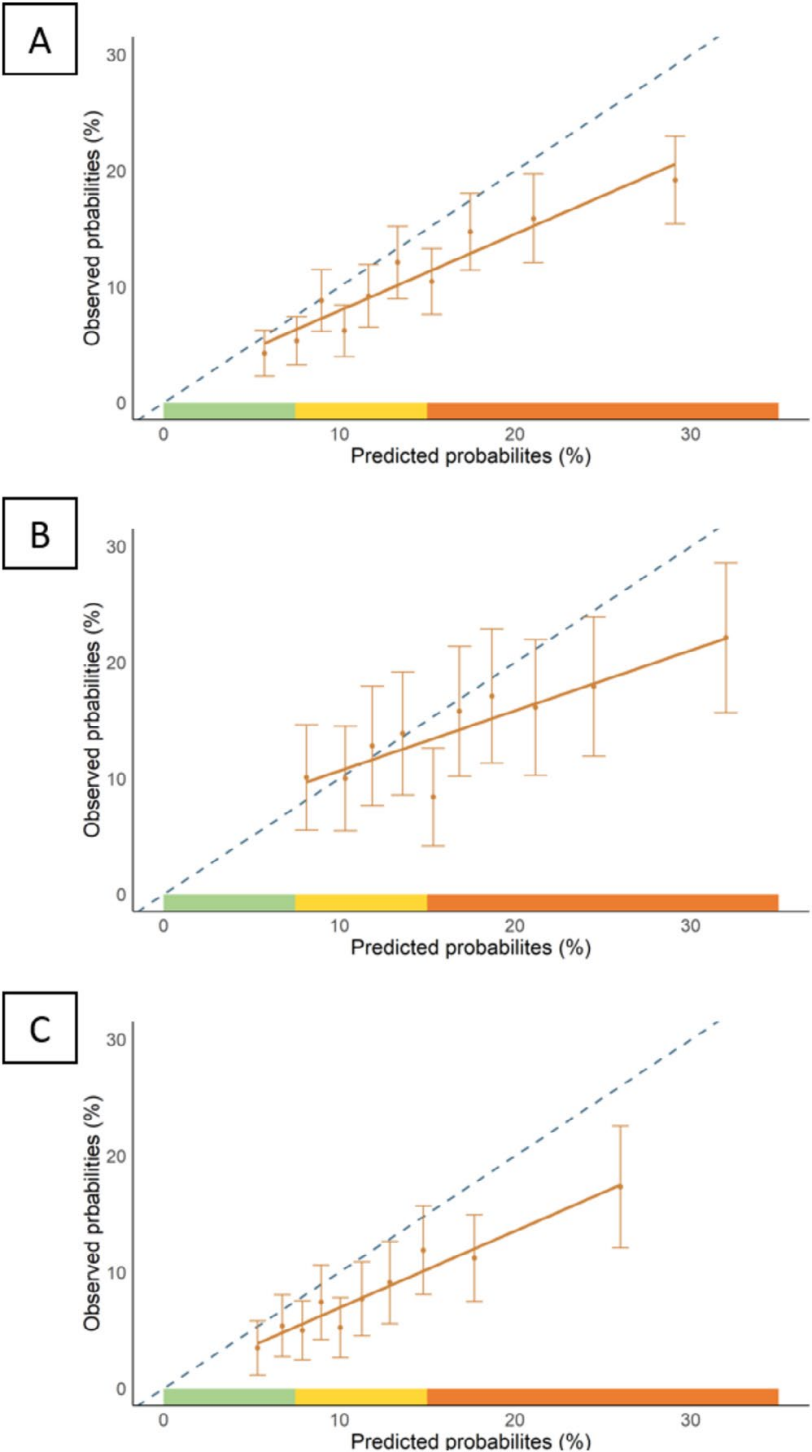
Calibration plots of predicted versus observed 10‐year cardiovascular risk in the overall population (*N* = 4626) [A], males (*N* = 1710) [B], and females (*N* = 2916) [C].

After adjustment for competing risks, the observed 10‐year cardiovascular event cumulative incidence was 10.55%, 95% CI = (9.62; 11.48), while SCORE2‐OP predicted risk was 14.04%, 95% CI = (13.85; 14.24) (Table S3), yielding an *O*/*E* ratio of 0.75, 95% CI = (0.69; 0.80), indicating 25% overestimation. This overestimation was greater in females (32%) than in males (17%) (Table S3, Figure 1).

Discrimination was moderate (10‐year AUC = 61.71%, 95% CI = [58.64; 64.78]), higher in females (AUC = 64.12%, 95% CI = [59.83; 68.40]) than in males (AUC = 57.73%, 95% CI = [53.16; 62.29]) (*p* = 0.04). AUCs at specific time points remained stable over time overall and in males, but declined in females (Figure S2).

### 3.3 Sleep Symptoms and Incidence of Cardiovascular Events

At baseline, 18.7% reported EDS. Among insomnia symptoms, 36.2% had DIS, 64.7% had DMS, and 37.8% had EMA. Additionally, 13.8% reported poor sleep quality. The median CSS score was 6 (range: 0–15). A potential sleep apnea syndrome was identified in 11.9% of participants, and 24.1% used hypnotics (17.4% benzodiazepines, 5.2% benzodiazepine‐like, 1.9% antihistaminic compounds, 1.6% sedative antidepressants, 1.5% other).

Poor sleep quality, DIS, DMS, EMA, CSS score, and potential sleep apnea were not associated with incident cardiovascular events after adjustment for cardiovascular risk factors and study center. Only EDS remained an independent predictor of cardiovascular events after full adjustment (Table 1). No interaction was found between sex and EDS for cardiovascular risk (*p* = 0.67).

**TABLE 1:** Associations between individual sleep symptoms and the risk of cardiovascular event over the 10‐year period.

	Cardiovascular event				Model 1		Model 2		Model 3	
	No (*N* = 4160)		Yes (*N* = 466)							
	*n*	%	*n*	%	HR (95% CI)	*p*	HR (95% CI)	*p*	HR (95% CI)	*p*
Excessive daytime sleepiness										
Never/rarely	3404	81.8	356	76.4	1	0.003	1	0.02	1	0.03
Frequent/often	756	18.2	110	23.6	1.4 (1.1; 1.7)		1.3 (1.1; 1.7)		1.3 (1.0; 1.6)	
Sleep quality										
Good/average	3194	85.9	373	88.8	1	0.09	1	0.30	1	0.24
Bad	526	14.1	47	11.2	0.8 (0.6; 1.1)		0.9 (0.6; 1.2)		0.8 (0.6; 1.1)	
Difficulty initiating sleep										
Never/rarely	2618	63.6	304	65.7	1	0.38	1	0.36	1	0.44
Frequent/often	1496	36.4	159	34.3	0.9 (0.8; 1.1)		1.1 (0.9; 1.4)		1.1 (0.9; 1.3)	
Difficulty maintaining sleep										
Never/rarely	1463	35.5	156	33.7	1	0.40	1	0.51	1	0.62
Frequent/often	2659	64.5	307	66.3	1.09 (0.9; 1.3)		1.1 (0.9; 1.3)		1.1 (0.9; 1.3)	
Early morning awakening										
Never/rarely	2540	61.9	298	64.9	1	0.20	1	0.82	1	0.63
Frequent/often	1567	38.1	161	35.1	0.88 (0.7; 1.1)		1.0 (0.8; 1.2)		1.0 (0.8; 1.2)	
Clinical sleep severity score										
Low/moderate (< 8)	2529	61.3	281	60.4	1	0.70	1	0.21	1	0.28
High (≥ 8)	1595	38.7	184	39.6	1.04 (0.9; 1.3)		1.1 (0.9; 1.4)		1.1 (0.9; 1.4)	
Proxy of sleep apnea syndrome										
No	3163	88.6	348	84.3	1	0.01	1	0.07	1	0.13
Yes	408	11.4	65	15.7	1.43 (1.1; 1.9)		1.3 (0.9; 1.7)		1.2 (0.9; 1.6)	

*Note*: Cox proportional hazard models: Model 1 was unadjusted. Model 2: each sleep symptom was adjusted for age, sex, SBP, total cholesterol, HDL‐c, current smoking, diabetes, study center. Model 3: each sleep symptom was adjusted for covariates included in model 2 plus lipid lowering and BP lowering medications.

Abbreviations: CI, confidence intervals; HR, hazard ratio.

### 3.4 Development of the SCORE2‐OP Incremented With EDS: SCORE2OP‐EDS

The sex‐specific coefficients of the linear predictor of the SCORE2‐OP‐EDS model along with the new cardiovascular risk equation are presented in Table S4. Additionally, the performances of SCORE2‐OP‐EDS are described in Figures S3 and S4.

Adding EDS to SCORE2‐OP did not improve overall discrimination (ΔAUC = +0.72%, 95% CI = [−0.05; 1.50], *p* = 0.07). In sex‐stratified analyses, a significant improvement was observed in males (ΔAUC = +2.27%, 95% CI = [0.54; 4.00], *p* = 0.01), but not in females (ΔAUC = −0.13%, 95% CI = [−0.64; 0.38], *p* = 0.61).

EDS improved reclassification of non‐events (NRI = +18.34%, 95% CI = [16.93; 19.75]) but worsened it for events (NRI = −17.01%, 95% CI = [−21.39; −12.64]), yielding a non‐significant overall NRI of +1.33%, 95% CI = (−3.27; 5.93). Sex‐specific analyses indicated consistent findings in females and non‐significant results in males (Tables 2, S5, S6).

**TABLE 2:** Reclassification of cardiovascular risk using SCORE2‐OP with and without excessive daytime sleepiness (EDS) in the overall study population.

10‐year CVD risk predicted by SCORE2OP	10‐year CVD risk predicted by SCORE2OP with EDS			Estimated risk increase	Estimated risk decrease	NRI for non‐events (95% CI)
	< 7.5%	(7.5; 15%)	≥ 15%			
Subjects without events during the follow‐up (*N* = 4160)						
< 7.5%	1297	125	0	3.28%	21.62%	18.34% (16.93; 19.75)
(7.5; 15%)	537	1523	11			
≥ 15%	0	364	303			
Subjects with events during the follow‐up (*N* = 466)						
< 7.5%	70	19	0	4.99%	22.00%	−17.01% (−21.39; −12.64)
(7.5; 15%)	36	201	5			
≥ 15%	0	65	70			
Overall net reclassification improvement (NRI) (95% CI)						1.33% (−3.27; 5.93)

Abbreviation: CVD, cardiovascular disease.

When stratifying cardiovascular risk (< 7.5%, 7.5%–15%, ≥ 15%), EDS improved reclassification in the low‐risk group (NRI = +11.99%, 95% CI = [3.56; 20.43]) but worsened it in the high‐risk group (NRI = +6.26%, 95% CI = [−2.80; 15.31]). In the intermediate‐risk group, EDS improved reclassification of non‐events (NRI = +25.42%, 95% CI = [23.49; 27.35]), but misclassified events (NRI = −12.98%, 95% CI = [−17.82; −8.14]), resulting in a positive overall NRI of +12.44%, 95% CI = (7.23; 17.65) (Table S7).

## 4 Discussion

In this French cohort of adults aged 70+ without prior CVD, the SCORE2‐OP model overestimated 10‐year cardiovascular risk by 25% and showed moderate discrimination (AUC = 61.71%). Adding EDS did not improve overall discrimination or reclassification. However, in the intermediate‐risk group, where preventive decisions are uncertain, improvements in non‐event reclassification compensated for events misclassification, yielding a significant overall NRI (NRI = +12.44%) and suggesting potential clinical relevance.

These findings align with prior external validations [5, 6, 7, 8, 9], showing AUCs between 51% and 69%. While EPIC‐Norfolk [8] and Barcelona cohorts [9] reported underestimations, we observed an overall overestimation, particularly in females. Several factors may explain these differences: our participants were older, predominantly female and more educated than those of the development cohort (CONOR). Lipid‐lowering and antihypertensive drugs were also more frequent (29% and 48% in 3C vs. ~0.6% and 28% in EPIC‐Norfolk), contributing to our cohort's lower CVD incidence (~10% vs. ~15% in EPIC‐Norfolk, 35% in CONOR). Additionally, excluded subjects were more often diabetic, a key CVD risk factor, which may have lowered the observed CV event rate. As SCORE2‐OP does not incorporate medication use, it may overestimate cardiovascular risk in treated individuals. In addition, France's relatively low rates of ischemic heart disease mortality, particularly among women [22], may have contributed to the overestimation.

Cohort recruitment may also explain differences. EPIC‐Norfolk and Barcelona cohorts enrolled primary healthcare patients (with potentially higher cardiovascular problems), whereas the 3C cohort was drawn from the general population. Additionally, lifestyle, healthcare access, regional care variations, and methodological differences (i.e., ICD code use, diagnostic definitions) may also contribute to disparities.

Furthermore, we assessed the incremental value of sleep symptoms as predictors of cardiovascular risk. Insomnia symptoms were not associated with incident cardiovascular event. This contrast with prior studies [13, 14, 15] may be due to the older age of our sample, where insomnia may be more a consequence than a determinant of CVD. In the absence of sleep recordings, we classified persons “at risk of obstructive sleep apnea syndrome” based on poor sleep quality, frequent EDS and loud snoring. After adjustment for cardiovascular risk factors, no association with cardiovascular incidence was found. Some of these factors such as diabetes and hyperlipidemia may overlap with sleep symptoms (e.g., snoring and poor sleep quality), reducing their independent contribution to predict cardiovascular risk. Furthermore, the low frequency of obesity in our sample, a key driver of sleep apnea, may have contributed to the lack of a significant association.

Among the sleep symptoms assessed in this study, only EDS was independently associated with CVD, consistent with prior publications [11, 12, 23, 24]. Intriguingly, adding EDS to SCORE2‐OP improved reclassification of non‐events (NRI = +18.34%) and simultaneously misclassified those with events (NRI = −17.01%), yielding a non‐significant risk reclassification. This downward reclassification could help reduce overtreatment in older adults with low cardiovascular risk [3]. The effect of EDS was particularly notable in the intermediate‐risk (7.5%–15%) category, where treatment decisions are often uncertain, suggesting a clinical interest for EDS in refining cardiovascular risk stratification in this subgroup.

Only a few studies have attempted to enrich the SCORE2‐OP. For comparison, adding chronic kidney disease markers to the SCORE2‐OP model yielded an overall NRI of 4% [25], mainly due to better reclassification of subjects with cardiovascular events (NRI = 9%) but poorer reclassification of subjects without events (NRI = −4%).

This study has limitations. Participants were volunteers with higher education and socio‐professional levels than the general population, limiting the generalizability of results. Sex‐specific analyses should be interpreted with caution given the loss of statistical power. EDS was self‐reported using a single question, which may introduce misperception. We also lacked data on sleep duration, timing, regularity, and apnea‐hypopnea index, factors known to influence cardiovascular risk [18, 19]. Finally, information on OSAS treatment (e.g., CPAP use) was unavailable and may have affected observed associations between sleep symptoms and cardiovascular events.

In conclusion, this study underlines the moderate performance of the SCORE2‐OP in predicting 10‐year cardiovascular risk in older adults and suggests that adding EDS did not significantly improve its overall performance. However, EDS may help refine risk reclassification in subjects with intermediate cardiovascular risk, the latter population for whom preventive decisions remain unclear. Further validations in low cardiovascular and high cardiovascular risk regions are also needed.

## Author Contributions

**Tahiana Andriambelosoa:** conceptualization, formal analysis, methodology, software, visualization, writing – original draft. **Clémence Cavaillès:** conceptualization, methodology, writing – review and editing. **Jean‐Philippe Empana:** conceptualization, methodology, writing – review and editing. **Thibault Mura:** methodology, writing – review and editing. **Catherine Helmer:** resources, writing – review and editing. **Yves Dauvilliers:** conceptualization, writing – review and editing. **Isabelle Jaussent:** methodology, conceptualization, software, supervision, writing – review and editing. All authors read and approved the final manuscript.

## Disclosure

Yves Dauvilliers participated in the advisory board for UCB Pharma, Jazz, Theranexus, Avadel, Idorsia, and Bioprojet, outside the submitted work.

## Conflicts of Interest

Yves Dauvilliers participated in the advisory board for UCB Pharma, Jazz, Theranexus, Avadel, Idorsia, and Bioprojet, outside the submitted work. The other authors declare no conflicts of interest.

## Supporting information


**Data S1:** Supporting Information.

## Data Availability

Deidentified participant data used in this study are available on reasonable request. Data access is subject to approval by the 3C Study Scientific Committee. Requests should be submitted to the corresponding author and 3C Study Scientific Committee. Additional information about the 3C Study, including study protocols and background documentation, is available at: https://the‐three‐city‐study‐3c.com.
