# Supplementary material for: SCORE2‐Older Persons (SCORE2‐OP): Validation and Added Value of Excessive Daytime Sleepiness in a French Cohort
Source: J Am Geriatr Soc. 2025 Aug 28;73(11):3519–25. doi: 10.1111/jgs.70047 (PMC12645540; doi:10.1111/jgs.70047)
Supplement: Supplementary file 1 — Data S1: Supporting Information. [file JGS-73-3519-s001.pdf]

SCORE2-OLDER PERSONS (SCORE2-OP): validation and added value of excessive daytime sleepiness in a French Cohort

SUPPLEMENTARY DATA

Supplement 1. Selection of the sample **p.2**

Supplement 2. Cardiovascular data collection, outcome assessment, and competing events **p.5**

Supplement 3. Predictors of cardiovascular events **p.6**

Supplement 4. Sleep symptoms and their definitions **p.6**

Supplement 5. Statistical Analysis **p.8**

Supplement 6. Results **p.10**

Supplement 1. Selection of the sample

The study sample included 4626 subjects aged 70 years and older, in line with the age range targeted by the SCORE2-OP model [1]. All participants were free of dementia, as cognitive impairment could affect their ability to complete sleep questionnaires. They also had no history of CVD, complete baseline data on SCORE2-OP predictors and EDS, and participated in at least one follow-up visit with information on incident cardiovascular events (Figure S1).

Figure S1. Flow chart of the sample.

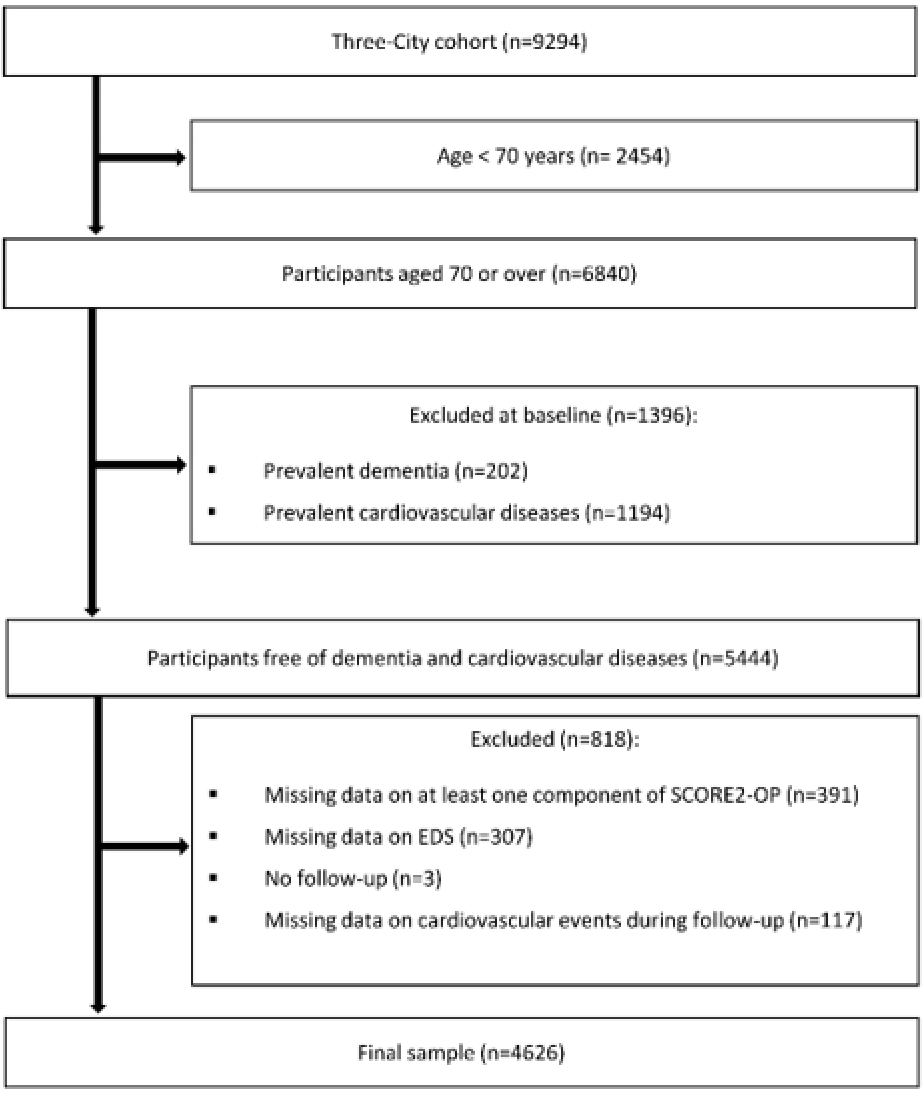

Table S1. Baseline demographic and clinical characteristics of the 4626 participants included in the final sample compared with the 818 excluded participants.

	Final sample (N=4626)	Excluded (N=818)	
**Characteristics**	**%**	**%**	**p-value[d]**
Sex, Female	63.04	67.73	0.01
Age (years) [a]	75.93 ± 4.39	77.83 ± 5.38	<0.0001
SBP (mmHg) [a]	148.33 ± 21.42	147.32 ± 23.00	0.24
Total cholesterol (mmol/L) [a]	5.86 ± 0.98	5.91± 1.03	0.31
Triglycerides (mmol/L) [a]	1.24± 0.58	1.27±0.62	0.04
HDL-c (mmol/L) [a]	1.63 ± 0.39	1.62 ± 0.42	0.86
Current smoking, yes	5.56	4.91	0.45
Diabetes, yes	8.54	18.07	<0.0001
Lipid-lowering drug, yes	28.82	25.55	0.05
Antihypertensive drug, yes	48.81	53.55	0.01
BMI class (kg/m²)	(n=4606)	(n=784)	0.74
<25	48.63	49.36	
[25 ;30[	38.97	37.63	
≥30	12.40	13.01	
Educational level (years)	(n=4618)	(n=814)	<0.0001
6	24.69	34.89	
[6 ;12[	35.55	34.40	
≥12	39.76	30.71	
CVD event at 10-year follow-up, yes	10.07	12.34	0.08
Risk category [c]	(n=4626)	(n=427)	<0.0001
<7.5%	32.66	21.08	
[7.5;15%[	50.00	52.93	
≥15%	17.34	26.00	
Excessive daytime sleepiness, yes	18.72	23.17	0.04

[a] Mean ± SD; [b] Median (Q1 ; Q3); [c] based on SCORE2-OP recalibrated with ratio O/E, [d] Student's t-test was used for continuous variables and Chi-squared test for qualitative variables.

Abbreviations: BMI= body mass index; SBP=Systolic Blood Pressure; HDL-c=High-density Lipoprotein Cholesterol, CVD=cardiovascular diseases

Compared to the included sample, the 818 participants excluded for missing data were more often female, older, diabetic, with lower triglycerides and educational level. They also had higher cardiovascular risk, took more antihypertensive medication and reported more frequently EDS (Table S1).

Supplement 2. Cardiovascular data collection, outcome assessment, and competing events

At baseline, participants provided information on their history of coronary heart disease (CHD), including angina pectoris or myocardial infarction, and stroke, by standardized clinical interviews.

At each follow-up visit, participants were asked to report any new severe medical events or hospitalizations for cardiovascular events since their last interview. For suspected cardiovascular events, further medical data were collected from general practitioners, specialists, and hospital records when possible.

Incident CHD events included hospitalized angina, myocardial infarction, coronary balloon dilatation, arterial bypass, and CHD death. All potential incident CHD cases were reviewed and validated by two independent experts. Stroke was defined according to the World Health Organization (WHO) criteria as a new focal neurological deficit of sudden or rapid onset of presumed vascular origin, lasting ≥24 hours. Each stroke case was confirmed by an adjudication committee to ensure accuracy [2,3].

Competing events were defined as non- cardiovascular deaths. Causes of death were collected by local study centers through medical records and interviews with family physicians, clinicians and other non-medical informants (relatives or caregivers). A validation committee used all information to classify the cause of death using the tenth revision of International Classification of Diseases (ICD). Exact dates of death were obtained from death registries.

Supplement 3. Predictors of cardiovascular events

Traditional CV risk factors were assessed at baseline using standardized evaluations. Demographic characteristics (age, sex) and smoking status (never or past user; current user) were recorded. Diabetes was defined as fasting glucose level ⩾7.0 mmol /L or current treatment. Systolic blood pressure (SBP) was measured as the mean of two measurements made on the right arm of the seated participant, using a digital tensiometer (OMRON M4). Antihypertensive treatment and lipid-lowering treatment (including statins) were documented and medications coded according to the WHO's Anatomical Therapeutic Chemical classification [4]. Lipid profile was assessed by measuring total cholesterol, triglycerides, and high-density lipoprotein (HDL) cholesterol levels.

Body mass index (BMI) was calculated from height and weight and categorized as: <25 kg/m2 (normal); [25–30[(overweight) and ≥30 (obese).

Supplement 4. Sleep symptoms and their definitions

Sleep symptoms were assessed at baseline by a self-administrated questionnaire. Participants were invited to answer the question "How would you assess your sleep?" (sleep quality) on a 3-point scale (0=good, 1=average, 2=bad) and to respond to other sleep questions using a 4-point Likert scale (0=never, 1=rarely, 2=frequently 3=often): "Do you feel very sleepy during the day?" (excessive daytime sleepiness, EDS), "Do you have any difficulty in falling asleep?" (difficulty initiating sleep, DIS), "Do you wake up during the night?" (difficulty maintaining sleep, DMS), "Do you often wake up early in the morning without being able to go back to sleep?" (early morning awakening, EMA), and "Do you snore loudly?". EDS and insomnia symptoms (e.g., DIS, DMS, EMA) were defined as reporting "frequently" or "often".

The combined effect of sleep quality, EDS and insomnia symptoms was evaluated using the Clinical Sleep severity (CSS) score, which quantifies the severity of sleep problems [5]. The sleep quality item

was recoded as 0= good, 1.5= average, and 3= poor. The total score was defined as the sum of the 5-item responses and range from 0 to 15. This score was categorized into “low and moderate score” *vs* “high score” based on the sample tertiles.

The likelihood of sleep apnea syndrome was defined as the report of frequent or often loud snoring together with EDS or poor sleep quality.

Supplement 5. Statistical Analysis

External validation of SCORE2-OP

We applied the SCORE2-OP calibrated for low-CVD risk regions to estimate 10-year cardiovascular risk, using the published intercept and regression coefficients [1]. Calibration is defined as the agreement between predicted and observed risks and discrimination as the model's ability to distinguish individuals at high risk from those at low risk. The observed cumulative incidence function (CIF) was estimated using the Aalen-Johansen method which accounts for competing risks (i.e. non-CV death). Expected risk was derived from the SCORE2-OP equations. As SCORE2-OP integrates sex-specific risk stratification, model's performances between males and females were compared to examine potential sex differences using a bootstrap method [6].

Sleep symptoms and cardiovascular events

We examined the association of each sleep symptom (i.e. poor sleep quality, EDS, insomnia symptoms, sleep apnea (proxy)) with incident cardiovascular events over 10 years using separate Cox proportional hazard models. Hazard ratios (HRs) and their 95% confidence intervals (CI) were estimated from univariable (model 1) and two multivariable models. Model 2 was adjusted for traditional cardiovascular risk factors considered in the SCORE-OP (i.e. age, SBP, HDL-c, total cholesterol, diabetes, smoking status) and study center, while model 3 was further adjusted for lipid-lowering and antihypertensive treatments. Only sleep symptoms significantly associated with cardiovascular events in model 3 were considered in the incremental value analysis (see below).

Added value of EDS to predict CVD with SCORE2-OP

From the above analysis, only EDS remained associated with incident cardiovascular events. First, we developed a sex-specific Fine and Gray model (SCORE2-OP-EDS), including the linear predictor of the original SCORE2-OP (calculated using the published intercept and regression coefficients [1]) and EDS considered as an additional predictor. Performances of this enriched model was assessed by evaluating both discrimination and calibration as described above.

Second, to evaluate the incremental value of EDS, we compared the AUC of the original SCORE2-OP model with the AUC of the extended model (SCORE2-OP-EDS). Additionally, survival net reclassification improvement (NRI) [7] was estimated across three pre-established cardiovascular risk categories: <7.5% (low risk); 7.5 to 15% (intermediate risk) and ≥15% (high-risk) [8]. The NRI assesses how well a new model improves risk classification compared to previous one, by quantifying 1) the net proportion of cases reassigned to a higher risk category minus those reassigned to a lower risk category, and 2) the net proportion of non-cases reassigned to a lower risk category minus those reassigned to a higher risk category. To address baseline risk differences in our cohort, we recalibrated the SCORE2-OP model by adjusting its probabilities using the cohort-specific O/E ratio [1] before estimating the NRI.

Statistical analyses were performed using R 4.4.1 statistical software particularly (R-packages: riskRegression, prodlim) and SAS version 9.4 (SAS Inc., Cary, NC, USA) (SAS macro: %PSHREG, %surv_nri).

Supplement 6. Results

Table S2. Baseline demographic and clinical characteristics of the 4626 participants compared with the development sample of SCORE2-OP (CONOR)[a].

**Cohort**	**3C (N=4626) (France)**	**CONOR (N=28503) (Norway)**	
**Recruitment period**	1999-2001	1994-2003	
**Characteristics**	**%**	**%**	**p-value [d]**
Sex, Female	63.04	50.00	<0.0001
Age (years) [b]	75.93 ± 4.39	73.00 ± 5.00	<0.0001
SBP (mmHg) [b]	148.33 ± 21.42	152.00 ± 23.00	<0.0001
Total cholesterol (mmol/L) [b]	5.86 ± 0.98	6.40 ±1.20	<0.0001
HDL-c (mmol/L) [b]	1.63 ± 0.39	1.50 ± 0.40	<0.0001
Current smoking, yes	5.56	20.00	<0.0001
Diabetes, yes	8.54	6.00	<0.0001
Lipid-lowering drug, yes	28.82	9.00	<0.0001
Cardiovascular event at 10-year follow-up, yes	10.07	35.00	<0.0001
Follow-up (years) [c]	9.18 (7.74 ; 9.73)	13.00 (8.00 ;15.00)	-

[a] Score2-OP working group and ESC Cardiovascular risk collaboration. SCORE2-OP risk prediction algorithms: estimating incident cardiovascular event risk in older persons in four geographical risk regions. *Eur Heart J* 2021;**42**:2455-2467.

[b] Mean ± SD ; [c] Median (Q1 ; Q3)

[d] Baseline characteristics of the 3C cohort were compared to those of the CONOR (SCORE2-OP development sample) using Student's t-tests for continuous variables and Chi-squared tests for categorical ones.

Abbreviations: SBP=Systolic Blood Pressure; HDL-c=High-density Lipoprotein Cholesterol

Table S3. Observed and expected 10-year cardiovascular (CV) events.

	Observed CV event [1]			Expected CV event[2]		Observed to Expected ratio [95 %CI]
	N participants	n events	% [95% CI]	n events	% [95%CI]	
Overall	4626	488	10.55 [9.62 ;11.48]	650	14.04 [13.85 ;14.24]	0.75 [0.69 ;0.80]
Male	1710	246	14.36 [12.65 ;16.07]	295	17.25 [16.98 ;17.57]	0.83 [0.73 ;0.92]
Female	2916	242	8.29 [7.23 ;9.36]	355	12.16 [11.98 ;12.36]	0.68 [0.60 ;0.76]

CI: confidence interval

(1) Competing fatal risk adjusted (Cumulative incidence)

(2) Given by the SCORE2-OP model

Figure S2. Area Under Curve (AUC) of SCORE2-OP at specific time points in the whole population (N=4626) [A], in males (N=1710) [B], in females (N=2916).

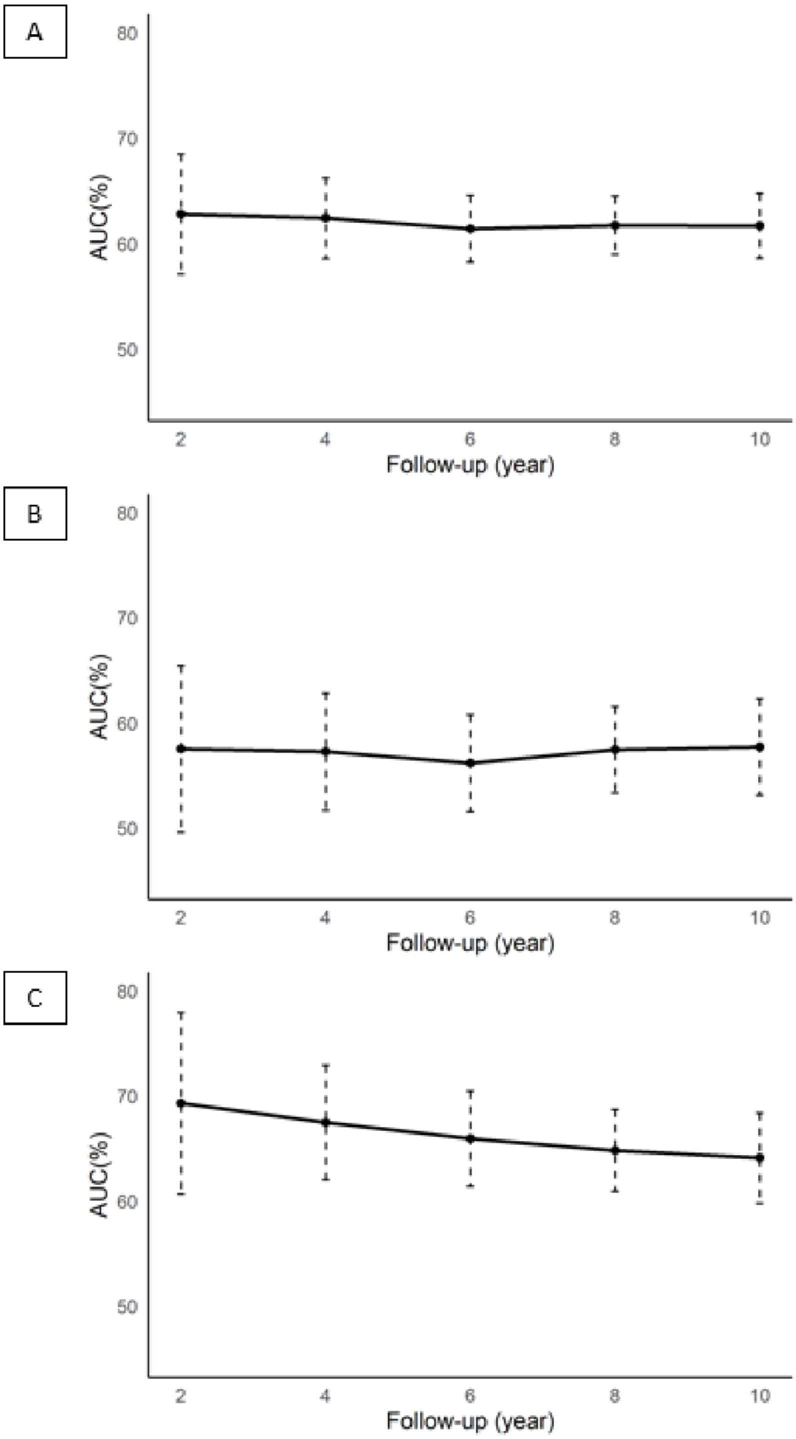

Table S4. Model SCORE2-OP-EDS: sex-specific coefficients of linear predictor (LP) of SCORE2-OP and excessive daytime sleepiness (EDS) for cardiovascular event in Fine and Gray model.

	$B_{SCORE2OP-EDS}$ [95% CI]	$LP_{SCORE2OP-EDS}$ equation	New cardiovascular risk equation
**In males (n=1710)**			
$LP_{SCORE2-OP}$	0.7143 [0.3541 ;1.0743]	0.7143 x $LP_{SCORE2-OP}$ + 0.2280x EDS	$1-(0.8839^{\,exp(LP_{SCORE2-OP-EDS}-0.1952)})$
EDS	0.2280 [-0.0544 ;0.5104]		
**In females (n=2916)**			
$LP_{SCORE2-OP}$	0.8683[0.6270 ;1.1092]	0.8683 x $LP_{SCORE2-OP}$ + 0.1276 x EDS	$1-(0.9339^{exp(LP_{SCORE2-OP-EDS}-0.1626})$
EDS	0.1276 [-0.2120 ;0.4669]		

Figure S3. Calibration plots for SCORE2-OP with EDS in the whole population (N=4626) [A], in males (N=1710) [B], in females (N=2916) [C].

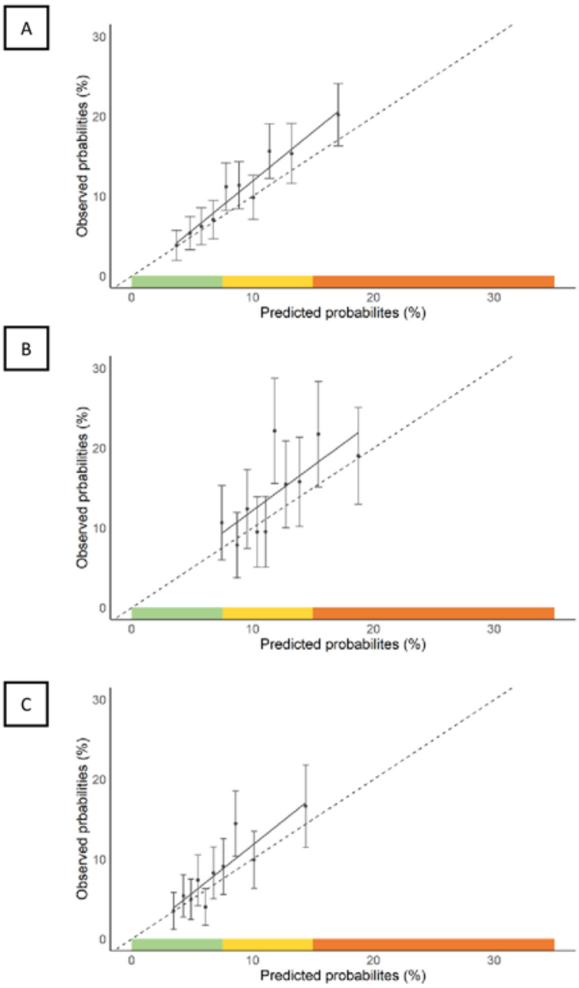

Calibration of the SCORE2OP-EDS showed an 18% underestimation of risk in the whole study population (O/E ratio=1.18, 95%CI=[1.08;1.26]), with similar results in males (O/E ratio=1.20, 95%CI=[1.05;1.33], 20% underestimation risk) and females (O/E ratio=1.16, 95%CI=[1.02;1.30], 16% underestimation risk) (Figure S3).

Figure S4. Area Under Curve (AUC) of SCORE2-OP with the addition of excessive daytime sleepiness at specific time-points in the whole study population [A], in males [B], in females [C].

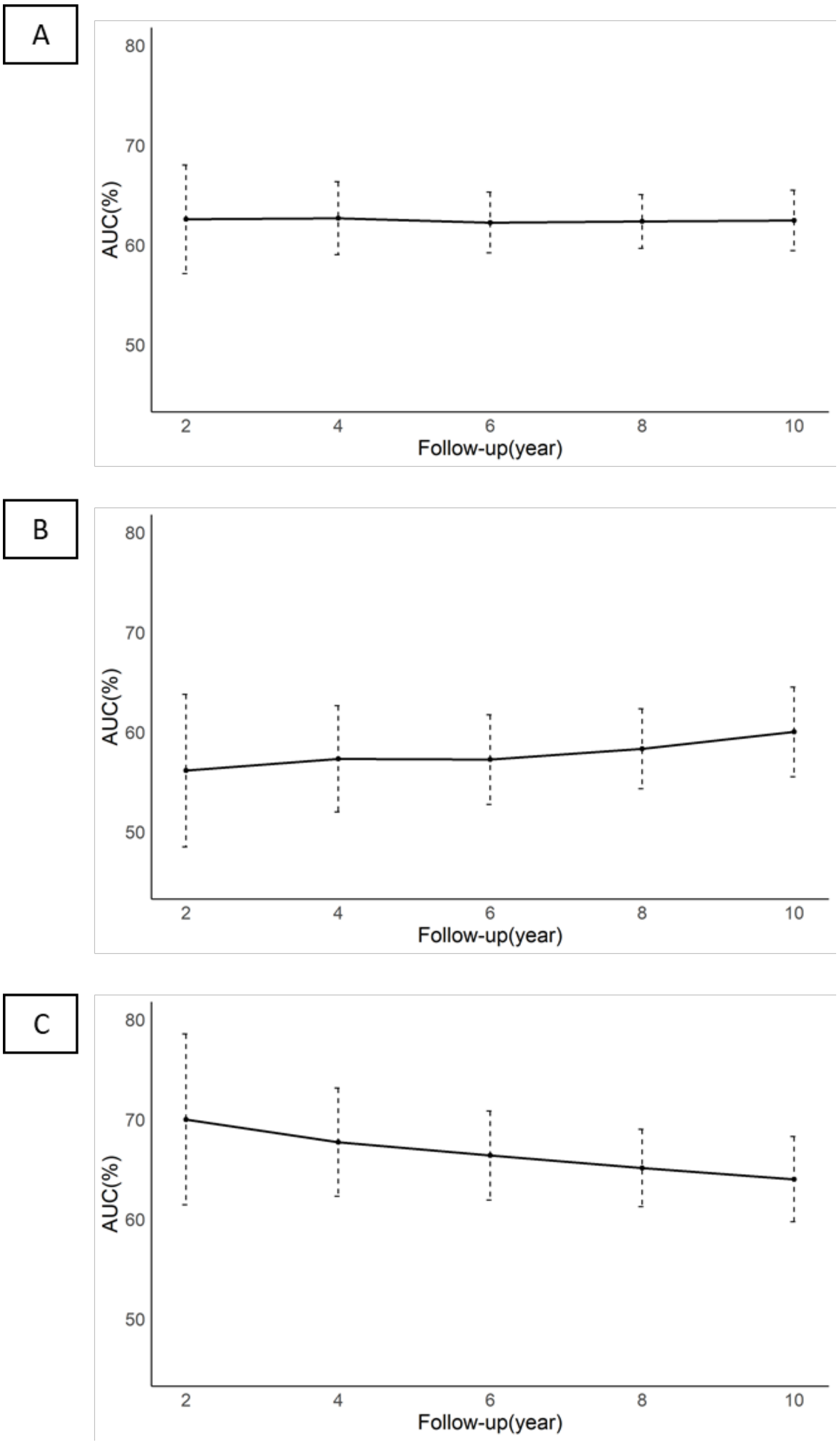

Discrimination was 62.43%, 95%CI=[59.40;65.46], with 63.99% in females (95%CI=[59.72;68.25]) and 60.0% in males (95%CI=[55.52;64.48]) (p for sex difference=0.19). Overall discrimination remained stable over time, whereas AUC decreased in females and increased slightly in males (Figure S4)

Table S5. Reclassification of cardiovascular risk using SCORE2-OP with and without excessive daytime sleepiness (EDS) in males.

**Subjects without events during the follow-up (n=1473)**						
	10-year CVD risk predicted by SCORE2-OP with EDS			Reclassification		
10-year CVD risk predicted by SCORE2-OP	<7.5%	[7.5 ;15%[	≥15%	Estimated risk increase	Estimated risk decrease	NRI for non-events [95% CI]
<7.5%	72	125	0			
[7.5 ;15%[	0	850	11	9.26%	13.18%	3.92% [1.50 ;6.33]
≥15%	0	194	221			
**Subjects with events during the follow-up (n=237)**						
10-year CVD risk predicted by SCORE2-OP						NRI for events [95% CI]
<7.5%	4	19	0			
[7.5 ;15%[	0	119	5	9.91%	14.25%	-4.34 [-10.49 ;1.81]
≥15%	0	34	56			
**Overall net reclassification improvement (NRI) [95% CI]**						-0.42 [-7.03;6.19]

Abbreviations: CVD=Cardiovascular disease

Table S6. Reclassification of cardiovascular risk using SCORE2-OP with and without excessive daytime sleepiness (EDS) in females.

	10-year CVD risk predicted by SCORE2OP with EDS			Reclassification		
**Subjects without events during the follow-up (n=2687)**						
10-year CVD risk predicted by SCORE2OP	<7.5%	[7.5 ;15%[	≥15%	Estimated risk increase	Estimated risk decrease	NRI for non-events [95% CI]
<7.5%	1225	0	0			
[7.5 ;15%[	537	673	0	0.00%	26.24%	26.24% [24.58 ;27.91]
≥15%	0	170	82			
**Subjects with events during the follow-up (n=229)**						
10-year CVD risk predicted by SCORE2OP						NRI for events [95% CI]
<7.5%	66	0	0			
[7.5 ;15%[	36	82	0	0.00%	29.89%	-29.89% [-35.69 ;-24.09]
≥15%	0	31	14			
**Overall net reclassification improvement (NRI) [95% CI]**						-3.65% [-9.68;2.39]

Abbreviations: CVD=Cardiovascular disease

Table S7. Reclassification of cardiovascular risk using SCORE2-OP with and without excessive daytime sleepiness (EDS) by cardiovascular risk stratification.

LOW CARDIOVASCULAR RISK SUBJECTS: <7.5%

Subjects without events during the follow-up (n=1422)

	10-year CVD risk predicted by SCORE2OP with EDS					
10-year CVD risk predicted by SCORE2OP	<7.5%	[7.5 ;15%[	≥15%	Estimated risk increase	Estimated risk decrease	NRI for non-events [95% CI]
<7.5%	1297	125	0	8.80%	0.00%	-8.80% [-10.28 ; -7.33]
[7.5 ;15%[	0	0	0			
≥15%	0	0	0			

Subjects with events during the follow-up (n=89)

	10-year CVD risk predicted by SCORE2OP with EDS					
10-year CVD risk predicted by SCORE2OP	<7.5%	[7.5 ;15%[	≥15%	Estimated risk increase	Estimated risk decrease	NRI for events [95% CI]
<7.5%	70	19	0	20.80%	0.00%	20.80% [12.49 ;29.10]
[7.5 ;15%[	0	0	0			
≥15%	0	0	0			

Overall net reclassification improvement (NRI) [95% CI] **11.99% [3.56;20.43]**

INTERMEDIATE CARDIOVASCULAR RISK SUBJECTS: [7.5%;15%[

Subjects without events during the follow-up (N=2071)

	10-year CVD risk predicted by SCORE2OP with EDS					
10-year CVD risk predicted by SCORE2OP	<7.5%	[7.5 ;15%[	≥15%	Estimated risk increase	Estimated risk decrease	NRI for non-events [95% CI]
<7.5%	0	0	0	0.54%	25.96%	25.42% [23.49 ;27.35]
[7.5 ;15%[	537	1523	11			
≥15%	0	0	0			

Subjects with events during the follow-up (N=242)

	10-year CVD risk predicted by SCORE2OP with EDS					
10-year CVD risk predicted by SCORE2OP	<7.5%	[7.5 ;15%[	≥15%	Estimated risk increase	Estimated risk decrease	NRI for events [95% CI]
<7.5%	0	0	0	1.94%	14.92%	-12.98% [-17.82 ; -8.14]
[7.5 ;15%[	36	201	5			
≥15%	0	0	0			

Overall net reclassification improvement (NRI) [95% CI] **12.44% [7.23;17.65]**

VERY HIGH CARDIOVASCULAR RISK SUBJECTS: ≥15%

Subjects without events during the follow-up (N=667)

	10-year CVD risk predicted by SCORE2OP with EDS					
10-year CVD risk predicted by SCORE2OP	<7.5%	[7.5 ;15%[	≥15%	Estimated risk increase	Estimated risk decrease	NRI for non-events [95% CI]
<7.5%	0	0	0	0	54.60%	54.60% [50.80 ;58.40]
[7.5 ;15%[	0	0	0			
≥15%	0	364	303			

Subjects with events during the follow-up (N=135)

	10-year CVD risk predicted by SCORE2OP with EDS					
10-year CVD risk predicted by SCORE2OP	<7.5%	[7.5 ;15%[	≥15%	Estimated risk increase	Estimated risk decrease	NRI for events [95% CI]
<7.5%	0	0	0	0	48.34%	-48.34% [-56.56 ; -40.13]
[7.5 ;15%[	0	0	0			
≥15%	0	65	70			

Overall net reclassification improvement (NRI) [95% CI] **6.26% [-2.80;15.31]**

Abbreviations: CVD=Cardiovascular disease

References supplementary data

1. SCORE2-OP working group and ESC Cardiovascular risk collaboration. SCORE2-OP risk prediction algorithms: estimating incident cardiovascular event risk in older persons in four geographical risk regions. *Eur Heart J*. 2021;42(25):2455-2467. doi:10.1093/eurheartj/ehab312

2. Blachier M, Dauvilliers Y, Jaussent I, et al. Excessive daytime sleepiness and vascular events: the Three City Study. *Ann Neurol*. 2012;71(5):661-667. doi:10.1002/ana.22656

3. Jaussent I, Empana JP, Ancelin ML, et al. Insomnia, Daytime Sleepiness and Cardio-Cerebrovascular Diseases in the Elderly: A 6-Year Prospective Study. *PLoS One*. 2013;8(2):e56048. doi:10.1371/journal.pone.0056048

4. World Health Organization. *World Health Organization Collaborating Centre for Drug Statistics Methodology: Guidelines for ATC Classification and DDD Assignment.*; 2000.

5. Cavaillès C, Berr C, Helmer C, Gabelle A, Jaussent I, Dauvilliers Y. Complaints of daytime sleepiness, insomnia, hypnotic use, and risk of dementia: a prospective cohort study in the elderly. *Alzheimers Res Ther*. 2022;14(1):12. doi:10.1186/s13195-021-00952-y

6. Robin X, Turck N, Hainard A, et al. pROC: an open-source package for R and S+ to analyze and compare ROC curves. *BMC bioinformatics*. 2011;12:77. doi:10.1186/1471-2105-12-77

7. Pencina MJ, D'Agostino RB, Steyerberg EW. Extensions of net reclassification improvement calculations to measure usefulness of new biomarkers. *Stat Med*. 2011;30(1):11-21. doi:10.1002/sim.4085

8. Visseren FLJ, Mach F, Smulders YM, et al. 2021 ESC Guidelines on cardiovascular disease prevention in clinical practice. *Eur Heart J*. 2022;23(6 Suppl 1):e3-e115. doi:10.1714/3808.37926
